# Mitochondrial ferritin in the regulation of brain iron homeostasis and neurodegenerative diseases

**DOI:** 10.3389/fphar.2014.00019

**Published:** 2014-02-17

**Authors:** Guofen Gao, Yan-Zhong Chang

**Affiliations:** Laboratory of Molecular Iron Metabolism, College of Life Science, Hebei Normal UniversityShijiazhuang, China

**Keywords:** mitochondrial ferritin, iron, brain, neurodegenerative diseases, oxidative damage

## Abstract

Mitochondrial ferritin (FtMt) is a novel iron-storage protein in mitochondria. Evidences have shown that FtMt is structurally and functionally similar to the cytosolic H-chain ferritin. It protects mitochondria from iron-induced oxidative damage presumably through sequestration of potentially harmful excess free iron. It also participates in the regulation of iron distribution between cytosol and mitochondrial contents. Unlike the ubiquitously expressed H-ferritin, FtMt is mainly expressed in testis and brain, which suggests its tissue-related roles. FtMt is involved in pathogenesis of neurodegenerative diseases, as its increased expression has been observed in Alzheimer’s disease, restless legs syndrome and Friedreich’s ataxia. Studies from our laboratory showed that in Alzheimer’s disease, FtMt overexpression attenuated the β-amyloid induced neurotoxicity, which on the other hand increased significantly when FtMt expression was knocked down. It is also found that, by maintaining mitochondrial iron homeostasis, FtMt could prevent 6-hydroxydopamine induced dopaminergic cell damage in Parkinson’s disease. These recent findings on FtMt regarding its functions in regulation of brain iron homeostasis and its protective role in pathogenesis of neurodegenerative diseases are summarized and reviewed.

## INTRODUCTION

Iron is an essential trace element for human health. In the brain, iron homeostasis is stringently regulated at three levels: organ, cellular, and subcellular, with different key regulatory molecules involved in each level. Dysregulation of brain iron homeostasis can lead to severe pathological changes in the neural system. For example, iron deficiency can slow down the development of neural system and cause language and motion disorders (Lozoff et al., 2000; Siddappa et al., 2004), while iron overload is closely related to neurodegenerative diseases (Zecca et al., 2004; Berg and Hochstrasser, 2006; Lee et al., 2006). The newly reported iron-storage protein, mitochondrial ferritin (FtMt) that locates in the mitochondria, possesses high homology to H-ferritin (Levi et al., 2001). It was reported that FtMt plays an important role in the regulation of cellular iron homeostasis (Corsi et al., 2002; Drysdale et al., 2002; Cazzola et al., 2003). Overexpression of FtMt affects iron homostasis and changes iron distribution between cytosol and mitochondria contents, and leads to cytosolic iron depletion (Corsi et al., 2002; Nie et al., 2005). In some neurodegenerative diseases characterized by iron overload, including Alzheimer’s disease and Parkinson’s disease (PD), increased expression of FtMt was observed (Shi et al., 2010; Wu et al., 2013). FtMt has a tissue-specific expression pattern and is rich in tissues with high metabolic activity, which is regarded as functionally important (Drysdale et al., 2002; Levi and Arosio, 2004). Evidences have shown that FtMt acts as a protective agent of neurons that maintains their normal functions and controls their apoptosis (Shi et al., 2010; Wang et al., 2011; Wu et al., 2013). Some of the molecular mechanisms underlying these protective functions were revealed recently, which provided insights into the pathogenesis of neurodegenerative diseases and may help the development of new therapeutic strategies.

## BRAIN IRON HOMEOSTASIS

Human bodies contain 3–5 g of iron in average. Dietary iron is absorbed predominately in duodenum and enters blood circulation in small intestine. Once in blood circulation, iron binds to apotransferrin and forms transferrin (Tf). Tf is the major vehicle for iron transport in the body, and carries iron to other cells and tissues through the circulation. At the target cell, Tf binds to transferrin receptors (TfR) on the cell membrane, and the TfR-Tf-Fe complex is then endocytosed into the cell, where the iron is released. Free iron either enters mitochondrion for utilization in metabolic processes, such as synthesis of hemoglobin and Fe-S cluster, or is incorporated into the cytosolic iron-storage protein, ferritin, and serves as a cellular store of iron.

Iron needs to pass the blood-brain barrier in order to enter the brain. Tf-Fe in the blood circulation is uptaken at the surface of cerebral capillary endothelia, mainly through the classic TfR-mediated endocytosis (Bradbury, 1997). In addition, TfR-independent Tf-Fe uptake may also exist. As shown by Ueda et al. (1993), non-TfR bound iron was transported into the brain when the TfR-mediated iron transport was maximally inhibited by anti-TfR antibodies. Free iron can also enter the brain barriers by divalent metal transport-1 (DMT1), a proton driven transporter (Siddappa et al., 2002; Skjorringe et al., 2012). In endothelia, iron is released and transported across the abluminal membrane of the barriers into the cerebral compartment. This process likely involves iron exporter ferroportin (FPN) and DMT1 on the abluminal membrane, but the exact mechanism remains for further exploration (Moos et al., 2007; Mills et al., 2010; Zheng and Monnot, 2012). The elemental iron released into the brain interstitial fluid binds to brain Tf and becomes available for neurons and neuroglia expressing TfR (Han et al., 2003). The excess iron in neurons and neuroglia can be exported back to the brain interstitial fluid, and can be released into the cerebrospinal fluid in the brain ventricles through bulk flow (Bradbury, 1997; Zheng and Monnot, 2012). The apical microvilli of choroidal epithelia then capture the free iron by TfR or DMT1 and transport it back to the blood circulation (Mills et al., 2010).

Iron homeostasis in brain is precisely regulated. At the cellular level, iron homeostasis is mainly regulated by iron transporters TfR, DMT1, and FPN. It has been reported that the uneven distribution of TfR in cerebral endothelia is responsible for the differences of iron concentrations in different brain regions (Deane et al., 2004). Iron concentrations are high in the striatum and the hippocampus where higher TfR density and iron uptake rate are also observed (Deane et al., 2004), but are low in the cortex and the brain stem (Morris et al., 1992; Sugawara et al., 1992). Similar to the iron regulation at the peripheral, iron homeostasis in brain is tightly regulated by iron regulatory proteins (IRPs) IRP1 and IRP2 (Rouault, 2006). When the brain cellular iron concentration is low, the active center of IRPs binds to the stem-loop structure of the iron-responsive element (IRE) located at the 3′-untranslated region (UTR) of TfR mRNA. This binding stabilizes TfR mRNA and increases its cellular expression level, thereby increasing iron uptake. When the iron concentration is high, the active center of IRP is occupied by four Fe-S, which blocks the binding of IRP to the IRE of TfR, resulting in low TfR translation level and reduced iron uptake (Leipuviene and Theil, 2007). The IRP/IRE system also regulates the stability of DMT1 with IRE (+IRE), FPN, and ferritin. However, binding of IRP to the IRE of FPN and ferritin decreases their stabilities, and causes lower protein expression (Rouault, 2006; Leipuviene and Theil, 2007). Thus, IRPs play a key role in the maintenance of cellular iron homeostasis. Studies of our laboratory and others have found that the IRP2^-^^/^^-^ mice had significant misregulation of iron metabolism and developed neurodegeneration (Meyron-Holtz, 2004). Inside the cells, the iron storage level and the cellular liable iron level (LIP) are largely dependent upon the availability of the iron-storage protein, ferritin. Ferritin is a ubiquitous protein with an iron core that can accommodate up to 4500 iron atoms (Theil, 1987; Harrison and Arosio, 1996). It is a 24-mer globular protein complex that is made up of heart (H) and liver (L) subunits, the H-ferritin (21 kDa) and the L-ferritin (19 kDa), respectively (Ford et al., 1984; Theil, 1987). The ability of ferritin to sequester iron provides its dual functions, iron segregation in a non-toxic form and iron storage (Harrison and Arosio, 1996; Torti and Torti, 2002).

At the systematic level, brain iron homeostasis may involve the regulation of an peptide “hormone” hepcidin (Crichton et al., 2011). Hepcidin is mainly produced by hepatocytes in response to high iron concentration, inflammatory stimuli or hypoxia (Park et al., 2001; Nicolas et al., 2002). It binds to the extracellular loop of FPN and causes its internalization and degradation, and thereby reduces cellular iron efflux (Ramey et al., 2010; Anderson and Wang, 2012). Several recent studies reported the identification of hepcidin producing cells in the brain and investigated hepcidin’s functions under normal and pathological conditions (Zechel et al., 2006; Marques et al., 2009; Wang et al., 2010; Crichton et al., 2011). Zechel et al. (2006) showed that hepcidin is widely expressed in different brain areas, including the cortex, hippocampus, thalamus, cerebellum, spinal cord, and so on, in both neurons and in GFAP-positive glia cells. Increased hepcidin expression was detected in choroid plexus of the brain in response to peripheral inflammation (Marques et al., 2009). Studies in our lab also found that hepcidin mRNA levels in different brain regions increased with aging, and injection of hepcidin into the lateral cerebral ventricle decreased FPN levels and resulted in brain iron overload (Wang et al., 2010). These findings implied the important regulatory role of hepcidin on brain iron metabolism, though the cellular mechanisms remain to be elucidated.

## MITOCHONDRIAL IRON HOMEOSTASIS AND MITOCHONDRIAL FERRITIN

### MITOCHONDRIAL IRON METABOLISM

Although most iron is stored in the cytosol, the major flux of iron in many cells occurs in the mitochondria, where various metabolic activities occur. Fe-S clusters and heme biogenesis are the main events in which iron is utilized (Ponka, 1997). Iron transport into mitochondria is directly coupled with its uptake at the cell membrane (Pandolfo, 2002; Levi and Rovida, 2009). Several mechanisms have been proposed on the pathway of iron entry into mitochondria. One hypothesis proposed by Ponka (1997) suggests that iron is directly delivered to mitochondria by endosomes in a “kiss and run” paradigm. Another theory proposed by Shvartsman et al. (2007) suggests that no endosomal vesicle is involved in the transport of non-Tf-bound iron to mitochondria. This was supported by the observation that mitochondrial iron uptake was not hampered by the use of cellular compartment-specific iron chelators, and chaperones were bound to the incoming iron prior to its delivery to micochondria (Shvartsman et al., 2007). Researchers also investigated the involvement of iron transport proteins on the mitochondrial membrane, such as MRS3 and MRS4 identified in yeast (Foury and Roganti, 2002) and Mitoferrin 1 and Mitoferrin 2 found in zebra fish (Paradkar et al., 2009). Iron transport out from mitochondria may depend on adequate Fe-S synthesis (Pandolfo, 2002).

Iron flux in mitochondria must be precisely regulated because excess free iron can result in the production of damaging free reactive oxygen species (ROS) during electron transport (Eaton and Qian, 2002). Dysregulation of mitochondrial iron metabolism can severely affect the intracellular iron homeostasis, resulting in mitochondrial iron metabolism diseases, such as Friedreich ataxia (FRDA; Schmucker and Puccio, 2010). However, little is known about the regulatory mechanisms of iron trafficking and communication between cytosol and mitochondria. It has been reported that ferritins, under the influence of iron and oxygen metabolism, exert cellular protective roles against iron-mediated free radical damage (Arosio and Levi, 2002; Arosio et al., 2009). The newly identified H-ferritin-like protein in mitochondria, FtMt, has been shown to modulate cellular iron metabolism and influence ROS level dramatically (Levi et al., 2001; Corsi et al., 2002; Nie et al., 2005). Studying the role of FtMt in mitochondria iron homeostasis may provide new insights into the treatment of diseases associated with abnormal iron homeostasis.

### FtMt SYNTHESIS AND DISTRIBUTION

Mitochondrial ferritin was first identified in 2001 as a new human ferritin type that specifically locates in mitochondria (Levi et al., 2001). Other primates, mice, and rats also express this gene, which is highly homologous to human FtMt. The human FtMt gene is intronless and locates at chromosome 5q23.1. It encodes a ~1 kb mRNA that translates to a 242 amino-acid FtMt precursor protein with a ~60 amino-acid mitochondrial targeting signal sequence at the N-terminus. The sequence of the mature human FtMt has a 79% identity to the H-chain ferritin. The ferroxidase centers of FtMt and H-ferritin share a completely conserved sequence and a fully overlapped crystallographic structure (Langlois d′Estaintot et al., 2004), indicating their similar functions. Recombinant FtMt was proven to have iron incorporation activity *in vitro* that was as efficient as H-ferritin (Bou-Abdallah et al., 2005). However, unlike the cytosolic ferritins, FtMt mRNAs lack the IRE consensus sequences for iron-dependent translational regulation.

The ~30 KDa human FtMt precursor protein is translocated to the mitochondria after synthesis, and is processed to become the ~22 KDa mature protein as the subunit to form typical ferritin shells (Corsi et al., 2002). Unlike the ubiquitously expressed cytosolic H-ferritin, the expression of FtMt is tissue-specific, showing a high level of transcription in testis and brain. Immunohistochemistry analyses of mouse FtMt showed its expression in spermatids and interstitial cells, neuronal cells of brain and spinal cord, and some other tissues. But surprisingly no expression was detected in hepatocytes, splenocytes, or myocytes (Drysdale et al., 2002; Levi and Arosio, 2004; Santambrogio et al., 2007). This further suggests that FtMt expression is not related to the cellular iron level, and the expression pattern may reflect its tissue-related roles. It was also found that, in the pathological conditions associated with mitochondrial iron overload, such as Alzheimer’s disease, PD, and sideroblastic anemia, the FtMt expression was largely induced (Cazzola et al., 2003; Shi et al., 2010; Wang et al., 2011; Wu et al., 2013; Yang et al., 2013).

### ROLE OF FtMt IN MITOCHONDRIAL AND CYTOSOLIC IRON DISTRIBUTION

As mentioned above, FtMt is structurally and functional similar to H-ferritin. The main biological function of FtMt is to incorporate excess free iron. It had a reduced ferroxidase activity as compared to H-ferritin, but the iron sequestering efficiency is as high (Corsi et al., 2002; Levi and Arosio, 2004). In addition to iron sequestration, FtMt was extensively studied on its function of maintaining intracellular iron homeostasis by modulating the traffick of iron in cytoplasm (Levi et al., 2001; Corsi et al., 2002; Nie et al., 2005). Corsi et al. (2002) found that overexpression of human FtMt in Hela cells resulted in decreased cytosolic ferritin and increased TfR levels and cytosolic iron deficiency. Using a stable cell line transfected with mouse *FtMt* gene, Nie et al. (2005) also observed that FtMt dramatically affected intracellular iron metabolism. Overexpression of FtMt caused an increase in cellular iron uptake but a decreased cytosolic iron level associated with decreased cytosolic ferritin, suggesting that the increased iron influx was preferentially transferred into mitochondria and incorporated into FtMt rather than into cytosol (Nie et al., 2005). They also found that the expression of FtMt was associated with decreased mitochondrial and cytosolic aconitase activities, which was consistent with the increase in IRP-IRE mRNA binding activity (Nie et al., 2005). In addition, increased expression of FtMt was found in some genetic diseases associated with cellular iron deficiency and mitochondrial iron overload, such as the restless legs syndrome (RLS; Ondo, 2005; Snyder et al., 2009). Many detailed advances in the research of FtMt and related diseases are summarized below.

## MITOCHONDRIAL FERRITIN IN THE PATHOPHYSIOLOGY OF NEURODEGENERATIVE DISEASES

### IRON, ROS AND CELL APOPTOSIS

Excess iron in brain is known to cause neurodegeneration in adults (Zecca et al., 2004). Increased ferrous iron (Fe^2^^+^) levels can lead to the production of highly reactive hydroxyl radical via the Fenton reaction. Increased iron levels can also generate peroxyl/alkoxyl radicals due to Fe^2^^+^-dependent lipid peroxidation (Pollitt, 1999). These ROS can damage cellular macromolecules including proteins, lipids and DNA, and finally the oxidative stress will trigger apoptosis. Iron-induced oxidative stress can be very destructive because a positive-feedback loop can develop from the release of more free iron from the iron-containing proteins, such as ferritin, heme proteins, and Fe-S clusters. As a result, the toxic effect of brain iron overload is exacerbated.

### FtMt IN THE PATHOPHYSIOLOGY OF PARKINSON’S DISEASE

**FIGURE 1:**
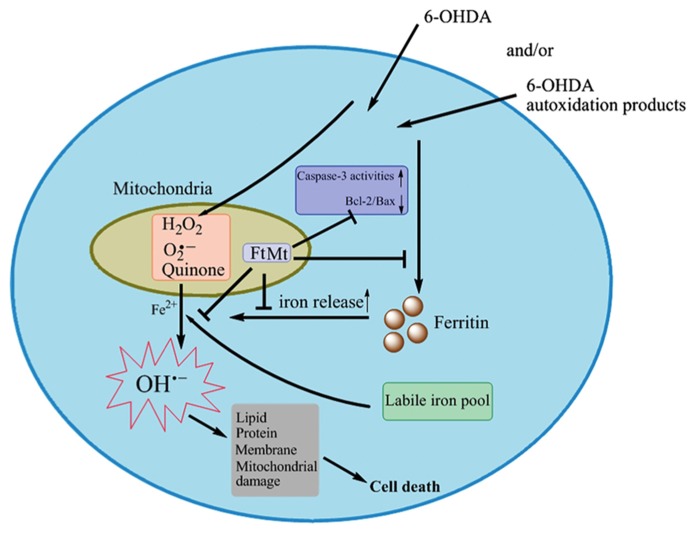
**A schematic representation of the proposed neuroprotective mechanism of FtMt in PD (adapted from Shi et al., 2010; Antioxid Redox Signal).** In the 6-OHDA-induced PD model, the overexpression of FtMt protects neuronal cells from programmed cell death. FtMt inhibits iron release from cytosolic ferritin, attenuates ROS accumulation and lipid peroxidation, and rescues LIP elevation induced by 6-OHDA, which in turn affects the Bcl-2, Bax and caspase-3 apoptotic signals, and prevents cell death.

Parkinson’s disease is a common neurodegenerative disease characterized by the loss of dopaminergic neurons in the substantial nigra (SN) of the brain and the formation of filamentous intraneuronal inclusions (Parkinson, 2002). The pathogenesis of PD involves accumulation of non-heme iron in the SN and nigra, oxidative damages and dysfunctions of mitochondria (Halliwell, 1992; Zhang et al., 2000; Zecca et al., 2004; Lin and Beal, 2006). Studies in our lab have shown that FtMt maintains iron homeostasis and prevents neuronal damage in a 6-Hydroxydopamine (6-OHDA)-induced parkinsonian phenotype (Shi et al., 2010). In our studies, the neuroblastoma SH-SY5Y cells were stably transfected with FtMt gene, and the PD model was established by induction with the neurotoxin 6-OHDA. We found that overexpression of FtMt significantly protected neuronal cells from the 6-OHDA-induced cell death. A possible mechanism of this protection was proposed which involves the regulation of Bcl-2, Bax, and caspase-3 apoptotic pathways (**Figure 1**). FtMt attenuated ROS accumulation and lipid peroxidation, and inhibited mitochondrial damage induced by neurotoxin 6-OHDA. Moreover, FtMt strongly inhibited the elevation of iron levels and prevented the alteration of iron redistribution induced by 6-OHDA. These findings suggest that FtMt plays a neuroprotective role in PD by affecting the iron metabolism.

### FtMt IN THE PATHOPHYSIOLOGY OF ALZHEIMER’S DISEASE

Alzheimer’s disease (AD) is a common neurodegenerative disease in aged people. The brains of AD patients are characterized by extracellular plaques of amyloid-β (Aβ) and neurofibrillary tangles of tau protein (Selkoe, 1996). Aβ plays an important role in the pathophysiological mechanisms of AD, as it accumulates to abnormally high levels in the brains of AD patients and directly induces neuronal cell death (Selkoe, 2000, 2001). Although abnormal iron metabolism and impaired mitochondrial function have been reported in AD, little information is available about the role of FtMt in the pathogenesis of AD.

A recent study by Wang et al. (2011) investigated the expression and localization of FtMt in the temporal cortex and cerebellum of AD patients. By using RT-PCR, they found that the FtMt mRNA levels in the temporal cortex of AD patients were evidently increased as compared to the controls, but no significant differences of mRNA levels was found in the cerebellum. By *in situ* hybridization histochemistry, FtMt mRNAs were localized mainly in the neurons of the AD cortex. They also found that in human neuroblastoma cell IMR-32, FtMt expression was significantly induced by H_2_O_2_ treatment, and the increase in FtMt expression was dramatically accelerated when cells were treated with the combination of H_2_O_2_ and Aβ neurotoxin. Overexpression of FtMt in the IMR-32 cells also rescued the cell death induced by H_2_O_2_. These results indicated a neuroprotection effect of FtMt against oxidative stress and the involvement of FtMt in the pathological process of AD. However, the underlying molecular mechanisms of FtMt’s action in AD and AD-like syndromes have not been fully elucidated.

To explore these mechanisms, our previous study by Wu et al. (2013) investigated the role of FtMt in Aβ25–35 treated rats. After the siRNA of FtMt was transfected into the hippocampus of the rats, we found that the FtMt down-regulated group released more cytochrome C, a sign of mitochondrial-dependent apoptosis, into the cytoplasm as compared to that of the control group. Increased number of apoptotic cells, decreased Bcl-2/Bax ratio and enhanced caspase-3 activation were observed, indicating a clear neuroprotectiove role FtMt plays *in vivo*. After treatment with Aβ25–35, knockdown of FtMt aggravated apoptosis in the hippocampus and oxidative damage to the tissue, as evidenced by increased levels of malonyl dialdehyde (MDA), protein carbonyls, and hydroxynonenal–histidine. The activities of the mitochondrial complex enzymes I–IV were also significantly decreased. To verify that the increased apoptosis was related to the low level of FtMt, we carried out further studies using SH-SY5Y cells that stably overexpressing FtMt. The results showed that FtMt overexpression reduced apoptosis in response to Aβ25–35 treatment and reduced the production of ROS as well. When FtMt was overexpressed in SH-SY5Y cells, the increase in caspase-3 protein and the reduction in the Bcl-2/Bax protein ratio following the Aβ25–35 treatment were largely neutralized. We further proposed that the direct neuroprotective effects of FtMt against Aβ25–35 toxicity could signal through the activation of the MAPK pathway in neurons, as the increase of extracellular signal regulated kinase (ErK) expression and the decrease of P38 level were observed.

**FIGURE 2:**
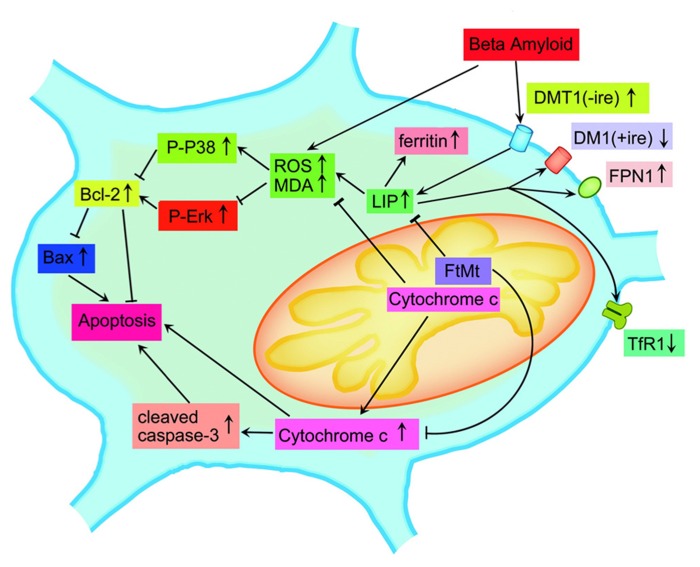
**A schematic representation of the proposed neuroprotective mechanism of FtMt in AD (adapted from Wu et al., 2013; Antioxid Redox Signal).** In the Aβ25–35-induced AD model, the overexpression of FtMt withdraws iron from the cytoplasmic pool, decreases LIP level, reduces oxidative damage through Erk/P38 kinase signaling, and prevents the release of cytochrome C into the cytoplasm. This in turn attenuates Aβ25–35-induced neurotoxicity and decreases the cell apoptosis through the MAPK pathway.

Evidences accumulated thus far have shown that iron metabolism is closely related to the production of oxidative stress and the pathogenesis of neurodegenerative diseases. We further determined the correlation of iron with the mechanism in which FtMt reduces ROS levels in the Aβ25–35-treated cells. In our study, FtMt overexpression dramatically inhibited the elevation of LIP levels resulted by the Aβ25–35 treatment. To verify that the change of LIP is involved in the protective function of FtMt, we measured the levels of iron related proteins. We observed that overexpression of FtMt increased the TfR level and decreased the H-ferritin level in Aβ25–35-treated cells (Shi et al., 2010; Wu et al., 2013). Without FtMt overexpression, these levels were measured to go the reverse way. These findings suggested that FtMt redistributed iron from the cytosol to the mitochondria, resulting in a reduction of cytosolic iron levels. This in turn attenuated Aβ25–35-induced neurotoxicity and reduced oxidative damage through the Erk/P38 kinase signaling. Our data also suggested that these effects were coordinately regulated by the intracellular LIP levels. Based on all these results, we proposed a possible neuroprotective mechanism of FtMt following the Aβ25–35 treatment, as shown in **Figure 2**.

### FtMt IN THE PATHOPHYSIOLOGY OF FRIEDREICH ATAXIA

Friedreich’s ataxia is the most common genetic ataxia that caused by the deficiency of mitochondrial iron-binding protein frataxin (Schmucker and Puccio, 2010). The FRDA patients have severe mitochondrial iron overload, disruption of iron-sulfur cluster biosynthesis, and increased sensitivity to oxidative stress (Schmucker and Puccio, 2010). The protective role of FtMt in FRDA was first suggested by Campanella et al. (2004) in a study on frataxin-deficient yeast cells. FtMt expression rescued the respiratory deficiency caused by the loss of frataxin and protected the activity of iron–sulfur enzymes in yeast. It also prevented yeast cells from developing mitochondrial iron overload, preserved the mitochondrial DNA integrity and increased resistance to H_2_O_2_. These data implied that FtMt could substitute most functions of frataxin in yeast, thus might play a protective role in FRDA. A follow-up study by Campanella et al. (2009) showed a similar function of FtMt in mammalian cells, including HeLa cells, and fibroblasts from FRDA patients. FtMt reduced the ROS level, increased the activity of mitochondrial Fe-S enzymes and the cell viability. Furthermore, FtMt expression reduced the LIP levels in both cytosol and mitochondria (Campanella et al., 2009). These results indicate that FtMt is involved in the regulation of iron distribution and availability in mitochondria and cytosol, thus controls ROS formation and protects cells characterized as defective in iron homeostasis and respiration.

### FtMt IN RESTLESS LEGS SYNDROME

Restless legs syndrome is a sensorimotor disorder. RLS patients are usually characterized as to have an urge to move the legs and to have abnormal sensations in the legs, especially in evenings and nights (Ondo, 2005). Unlike other neurodegenerative diseases, RLS was reported to have decreased cellular iron concentration in the brain and altered expression of iron metabolism-related proteins. Significant iron deficiency was observed in the neurons of SN in RLS patients (Connor et al., 2003; Schmidauer et al., 2005; Godau et al., 2007), and decreased ferritin and TfR and increased Tf were also observed, attesting the cellular iron deficient status (Connor et al., 2004). Considering the important role of iron in the redox reactions in mitochondria, Snyder et al. (2009) studied the expression pattern of FtMt in the brain of RLS patients. The results showed that the staining of FtMt increased significantly in the RLS cases, and the neuromelanin-containing neurons in the SN were found to be the predominant cell type expressing FtMt. Since the numbers of mitochondria were also increased in the neurons, whether the increase of FtMt was a result of higher FtMt expression or from mitochondrial proliferation with normal amounts of FtMt could not be concluded. However, less cytosolic H-ferritin were observed in neurons of RLS cases, suggesting that the increased FtMt levels might contribute to the insufficient cytosolic iron levels in the SN neurons, thereby accelerating the pathogenesis of RLS (Snyder et al., 2009). Still, very little is known about the metabolic activity of SN and the role of FtMt in RLS, and further investigations are needed to understand more on the mechanisms.

## SUMMARY

Mitochondrial ferritin is a novel ferritin type that specifically locates in mitochondria. It is highly expressed in tissues with high metabolic activity and oxygen consumption, such as testis, brain, heart, and so on. This tissue specificity may correlate with its function. Studies so far suggest that FtMt plays a role in the protection of mitochondria from iron-dependent oxidative damage by sequestering the free excess iron. Current findings suggested important roles of FtMt in the pathogenesis of neurodegenerative diseases. The increased expression of FtMt in AD, PD, and other neurological disorders may relate to its neuroprotective role against iron overload and oxidative stress. But in RLS, its increased expression may link to the onset of disease rather than neuroprotection. Since FtMt lacks the IRE in its mRNA, which is different from other ferritins, it should not be regulated by iron directly. Further studies regarding the detailed mechanisms of the regulation of FtMt expression and the role FtMt plays in neurological disorders associated with abnormal iron metabolism are important topics that need to be explored in the future.

## AUTHOR CONTRIBUTIONS

Yan-Zhong Chang conceived the review and participated in design and discussion; Guofen Gao drafted the manuscript and participated in discussion. All authors read and approved the final manuscript.

## Conflict of Interest Statement

The authors declare that the research was conducted in the absence of any commercial or financial relationships that could be construed as a potential conflict of interest.
